# Beyond Digoxin Toxicity: A Fatal Case of BRASH Syndrome Triggered by Community-Acquired Pneumonia

**DOI:** 10.7759/cureus.116978

**Published:** 2026-09-26

**Authors:** Ahmed Elnour, Wajeeh Sohail, Salahuddin Khan, Sebastian Urruela

**Affiliations:** 1 Internal Medicine, University Hospital Kerry, Tralee, IRL; 2 Internal Medicine, Mercy University Hospital, Cork, IRL; 3 Oncology, University Hospital Kerry, Tralee, IRL

**Keywords:** acute kidney injury, bradycardia, brash syndrome, digoxin, hyperkalemia

## Abstract

Bradycardia, renal failure, atrioventricular (AV) nodal blockade, shock, and hyperkalemia (BRASH) syndrome is a rare, life-threatening clinical condition caused by the synergistic interaction between hyperkalemia, renal dysfunction, and AV nodal blocking agents, resulting in severe bradycardia and hemodynamic instability. Early recognition is often challenging due to its nonspecific presentation.

We report the case of an 82-year-old man with atrial fibrillation, ischemic heart disease, congestive cardiac failure, chronic kidney disease, and chronic digoxin therapy who presented with abdominal pain, confusion, dizziness, hypotension, and profound bradycardia. Investigations demonstrated severe hyperkalemia, acute kidney injury on top of chronic kidney disease, hyponatremia, and electrocardiographic changes consistent with hyperkalemia. Initial differential diagnoses included mesenteric ischemia, adrenal insufficiency, and digoxin toxicity; however, imaging excluded bowel ischemia, cortisol levels were normal, and serum digoxin at the therapeutic level.

Community-acquired pneumonia complicated by sepsis was identified as the precipitating trigger for BRASH syndrome. Despite intensive treatment, including hyperkalemia management, isoprenaline infusion, antibiotics, corticosteroids, and supportive care, the patient’s condition progressively deteriorated, culminating in multiorgan failure and death shortly afterward.

This case highlights the diagnostic complexity of BRASH syndrome and emphasizes the importance of early recognition and targeted management to interrupt the vicious cycle and improve clinical outcomes.

## Introduction

BRASH syndrome, which stands for bradycardia, renal failure, atrioventricular nodal blockade (AVNB), shock, and hyperkalemia, is a recognized clinical entity first described by J. Farkas in 2016 [1,2]. It is characterized by the synergistic interaction between hyperkalemia and atrioventricular (AV) nodal blocking agents, resulting in profound bradycardia and hemodynamic instability [1,3]. The pathophysiological hallmark of BRASH syndrome is characterized by a vicious cycle involving renal failure and hyperkalemia, which is a leading cause of AV nodal block that leads to reduced cardiac output and eventually leads to reduced renal perfusion and worsening renal function, perpetuating the vicious cycle and potentially leading to rapid clinical deterioration if not recognized and treated promptly [2-4]. Beta-blockers, calcium channel blockers (mainly non-dihydropyridine), amiodarone, and digoxin are commonly implicated in BRASH syndrome. These atrioventricular nodal blocking agents are widely prescribed for cardiovascular conditions, such as hypertension, coronary artery disease, heart failure, and tachyarrhythmias; however, in susceptible patients, particularly those with renal impairment, they may precipitate BRASH syndrome [3,5].

Management of BRASH syndrome focuses on interrupting the vicious cycle through prompt correction of hyperkalemia, hemodynamic support, and withdrawal of contributing medications. In severe or refractory cases, temporary pacing or renal replacement therapy might be required [6,7]. Early recognition and targeted management are essential to prevent rapid clinical deterioration and multiorgan failure.

## Case presentation

An 82-year-old man presented with a three-day history of abdominal pain, confusion, and dizziness. His background was significant for type 1 diabetes mellitus, atrial fibrillation, ischaemic heart disease, congestive cardiac failure, hypertension, Parkinson's disease, and chronic lower limb ulceration. His regular medications included apixaban, amlodipine, insulin, carbidopa/levodopa, and digoxin. On arrival, he was pale, nauseated, and confused, with a temperature of 38.4 degrees Celsius. He was hypotensive, with a blood pressure of 80/60 mmHg, and profoundly bradycardic, with a heart rate ranging from 30 to 38 beats per minute. His respiratory rate was 18 breaths per minute, with an oxygen saturation of 92% on room air. Abdominal examination revealed distension without tenderness, and bowel sounds were preserved. Auscultation of the chest demonstrated right basal crackles consistent with a diagnosis of pneumonia, which was subsequently confirmed radiologically. The initial working diagnosis was mesenteric ischemia in the context of atrial fibrillation, with concurrent suspicion of digoxin toxicity in addition to community-acquired pneumonia. A 12-lead electrocardiograph (ECG) demonstrated marked bradycardia with tall, peaked T waves, raising concern for hyperkalemia (Figure 1). Initial laboratory investigations revealed hyperkalemia, high C-reactive protein (CRP) level, which reflects a current infection or inflammatory process (Table 1).

**Figure 1:**
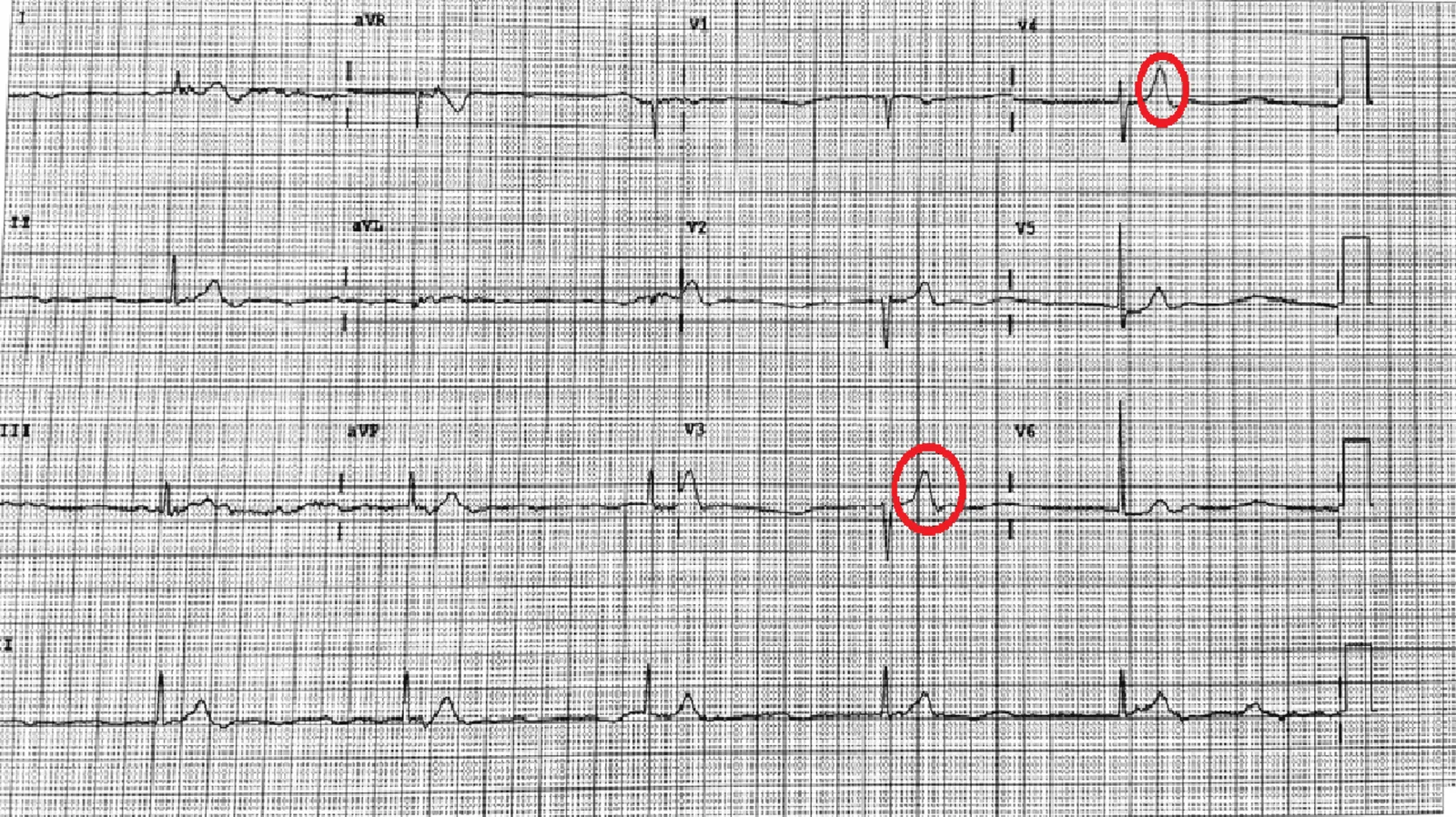
A 12-lead electrocardiograph (ECG) Bradycardia with tall, peaked T waves (circled), ECG signs of hyperkalemia.

**Table 1:** Full blood count (FBC) and basic metabolic panel Admission laboratory investigations. Laboratory results obtained at the time of presentation, demonstrating severe hyperkalemia, hyponatremia, acute kidney injury, and elevated inflammatory markers.

Parameter	Result	Reference range
White cell count	10 × 10⁹/L	4.0-11.0
Neutrophils	9 × 10⁹/L	2.0-7.5
Lymphocytes	0.32 × 10⁹/L	1.0-4.0
Haemoglobin	8.5 g/dL	13.0-17.0
Mean corpuscular volume	91 fL	80-100
Platelets	250 × 10⁹/L	150-400
Sodium	121 mmol/L	135-145
Potassium	7.2 mmol/L	3.5-5.0
Chloride	91 mmol/L	95-108
Urea	30 mmol/L	2.5-7.8
Creatinine	442 µmol/L	60-110
Estimated glomerular filtration rate	11 mL/min/1.73 m²	>60
Total protein	59 g/L	60-80
Albumin	33 g/L	35-50
Calcium	2.26 mmol/L	2.20-2.60
Phosphate	2.19 mmol/L	0.80-1.50
Magnesium	0.94 mmol/L	0.70-1.00
C-reactive protein	90 mg/L	<5
Free thyroxine	13.9 pmol/L	9-25
Thyroid-stimulating hormone	1.11 mIU/L	0.4-4.0
B-type natriuretic peptide	6000 pg/mL	<100
Random serum cortisol	700 nmol/L	>300nmol/L
Serum digoxin level	0.81 µg/L	0.8-2.0 (therapeutic)

The combination of hyponatremia, hyperkalemia and hypotension initially raised concern for adrenal insufficiency. Intravenous hydrocortisone was commenced after obtaining a random serum cortisol sample. The cortisol level subsequently returned at 700 nmol/L, making adrenal insufficiency unlikely. Notably, the serum digoxin concentration was within the therapeutic range (0.81 µg/L), making isolated digoxin toxicity an unlikely explanation for the profound bradycardia.

A computed tomography (CT) scan of the thorax and abdomen (Figure 2A, 2B) was performed to evaluate for mesenteric ischemia. The scan excluded bowel ischemia but demonstrated bilateral pleural effusions and right lung atelectasis consistent with community-acquired pneumonia. The overall clinical and biochemical findings supported sepsis secondary to community-acquired pneumonia as the precipitating event, leading to acute kidney injury on a background of chronic kidney disease stage 3a, with subsequent hyperkalemia and decompensated heart failure.

**Figure 2:**
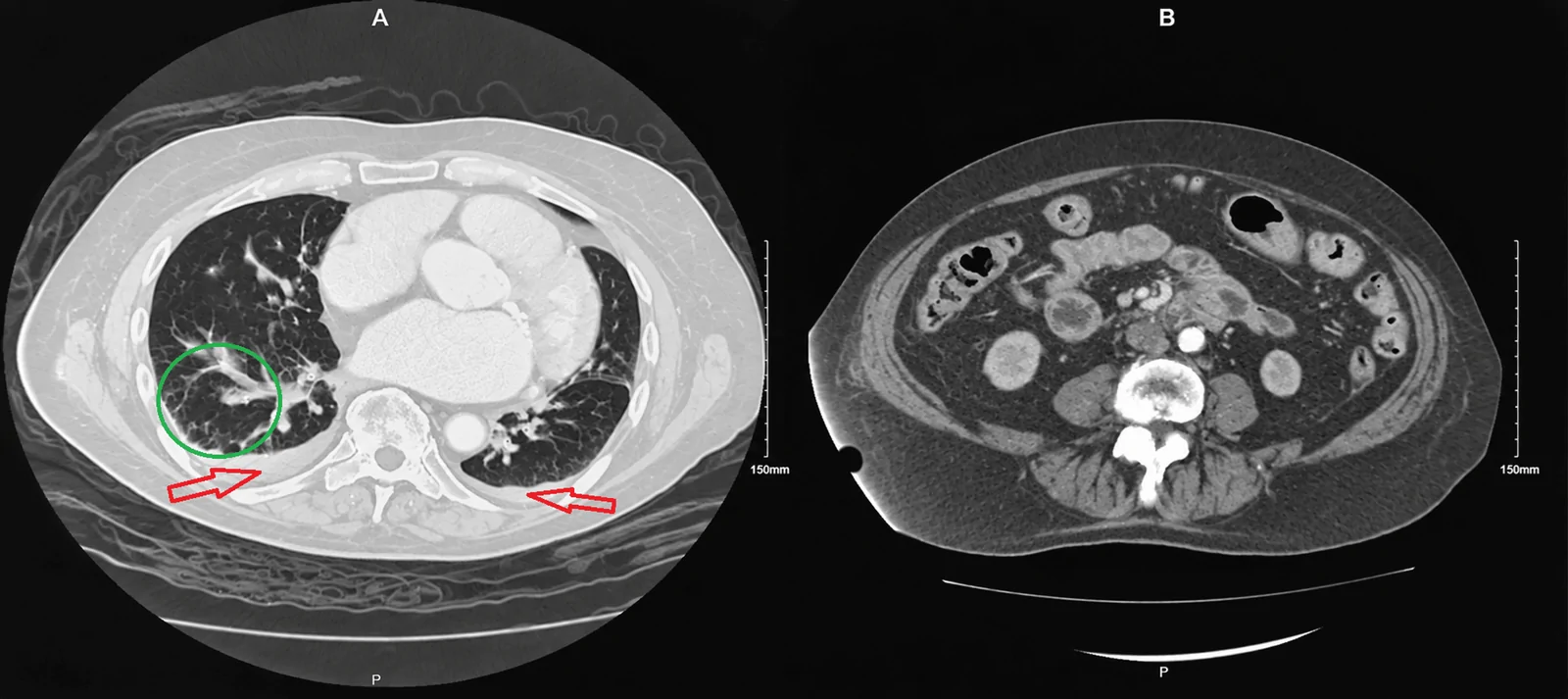
Computed tomography (CT) images of the thorax and abdomen. (A) CT image of the thorax demonstrating bilateral pleural effusions (red arrows) and associated right basal atelectatic changes (green circle). (B) Axial CT image of the abdomen showing no acute intra-abdominal abnormality.

The constellation of bradycardia, renal failure, AV nodal blockade (digoxin), shock, and hyperkalemia - occurring despite a therapeutic digoxin level - was diagnostic of BRASH syndrome, with community-acquired pneumonia identified as the likely precipitating trigger.

Given the profound bradycardia, hemodynamic instability, severe hyperkalemia, and acute kidney injury, the patient was admitted to the high dependency unit (HDU) for close monitoring and further management. Digoxin, amlodipine, and other potentially contributing medications were discontinued immediately on admission, he subsequently became anuric and was treated with the standard hyperkalemia protocol, including intravenous calcium gluconate, insulin-dextrose, and nebulized salbutamol. An isoprenaline infusion was commenced for symptomatic bradycardia, alongside intravenous hydrocortisone and antibiotics for community-acquired pneumonia. The patient was reviewed by the nephrology team; however, given his frailty, poor baseline performance status, and overall clinical condition, renal replacement therapy was considered inappropriate. Despite maximal supportive management, he continued to deteriorate, developing progressive multiorgan failure, and subsequently died later that day.

## Discussion

BRASH syndrome is a rare but potentially life-threatening clinical entity characterized by the coexistence of bradycardia, renal failure, atrioventricular nodal blockade, shock, and hyperkalemia. The syndrome results from a vicious cycle in which hyperkalemia and AV nodal blocking agents act synergistically to produce profound bradycardia, leading to worsening renal hypoperfusion and further metabolic derangement [1,2]. Acute illnesses, including infection, have been described as potential precipitating factors for BRASH syndrome [6]. In our patient, community-acquired pneumonia complicated by sepsis was present at presentation and may have contributed to the development of acute kidney injury and the subsequent BRASH physiology.

The diagnosis of BRASH syndrome can be challenging because its presentation frequently overlaps with isolated hyperkalemia, AV nodal blocker toxicity, septic shock, or primary cardiac conduction disorders [2,3,6].

In our patient, the initial differential diagnosis included mesenteric ischemia, digoxin toxicity, and adrenal insufficiency. The presence of severe hyperkalemia, acute kidney injury, profound bradycardia, and shock in a patient receiving chronic digoxin therapy raised suspicion for BRASH syndrome.

Although therapeutic concentrations of AV nodal blocking agents rarely cause profound bradycardia, the coexistence of renal dysfunction and hyperkalemia can synergistically potentiate their electrophysiological effects, resulting in severe bradyarrhythmia and conduction disturbances that are disproportionate to either factor alone [2,6,8]. Notably, the serum digoxin concentration was within the therapeutic range, emphasizing the distinctive pathophysiology of BRASH syndrome, where the combined effects of hyperkalemia and AV nodal blockade can produce profound bradycardia disproportionate to either insult alone [4,8]. Several factors likely contributed to the development of BRASH syndrome in this case. Community-acquired pneumonia resulted in sepsis and renal hypoperfusion, precipitating acute kidney injury on a background of chronic kidney disease. The resulting reduction in renal clearance promoted potassium retention and enhanced the physiological effects of chronic AV nodal blockade. Although digoxin was the principal AV nodal blocking agent, amlodipine may also have contributed to the patient's hemodynamic instability. The coexistence of chronic cardiovascular disease, renal impairment, and advanced age further increased susceptibility to this syndrome.

Management of BRASH syndrome requires simultaneous treatment of all components of the syndrome rather than focusing solely on bradycardia or hyperkalemia. Standard therapy includes withdrawal of AV nodal blocking agents, aggressive correction of hyperkalemia, optimisation of volume status, and treatment of the precipitating cause [1,6]. Vasopressor or chronotropic support may be required for refractory bradycardia, while severe cases may necessitate temporary pacing or renal replacement therapy [1,6,9].

In our patient, hyperkalemia was treated promptly with calcium gluconate, insulin-dextrose, and nebulized salbutamol, while isoprenaline infusion was used to support heart rate and perfusion. Empirical antibiotics were initiated for the underlying community-acquired pneumonia. Despite these measures, the prognosis was adversely influenced by several factors, including advanced age, significant cardiovascular comorbidity, chronic kidney disease with superimposed acute kidney injury, sepsis, profound bradycardia with hemodynamic instability, and subsequent anuria. Furthermore, renal replacement therapy was considered inappropriate because of his frailty and poor baseline functional status. These factors likely contributed to progressive multiorgan dysfunction and ultimately death.

## Conclusions

BRASH syndrome is a rare but potentially fatal condition resulting from the synergistic interaction between renal dysfunction, hyperkalemia, and AV nodal blockade. This case demonstrates how an acute infection such as community-acquired pneumonia and sepsis can precipitate acute kidney injury and likely trigger the BRASH cycle in a susceptible patient with underlying renal and cardiovascular disease. Importantly, profound bradycardia and hemodynamic instability may occur despite therapeutic levels of an AV nodal blocking agent, making the diagnosis challenging and potentially leading to confusion with isolated drug toxicity or hyperkalemia. Clinicians should consider BRASH syndrome when bradycardia, renal impairment, hyperkalemia, and AV nodal blocker use coexist. Early recognition, simultaneous correction of the underlying abnormalities, and treatment of the precipitating cause are essential to interrupt the vicious cycle and potentially improve outcomes.
